# Using Quality Measures for Quality Improvement: The Perspective of Hospital Staff

**DOI:** 10.1371/journal.pone.0086014

**Published:** 2014-01-23

**Authors:** Asgar Aghaei Hashjin, Hamid Ravaghi, Dionne S. Kringos, Uzor C. Ogbu, Claudia Fischer, Saeid Reza Azami, Niek S. Klazinga

**Affiliations:** 1 Department of Social Medicine, Academic Medical Center (AMC)/University of Amsterdam, Amsterdam, the Netherlands; 2 Department of Health Services Management, School of Health Management and Information Sciences, Iran University of Medical Sciences, Iranian Ministry of Health and Medical Education, Tehran, Iran; 3 Health Management and Economic Research Center, School of Health Management and Information Sciences, Iran University of Medical Sciences, Iranian Ministry of Health and Medical Education, Tehran, Iran; 4 Department of Public Health, Erasmus Medical Center, Rotterdam, the Netherlands; 5 Development and Supervision Management of Public Affairs, Tehran University of Medical Sciences, Iranian Ministry of Health and Medical Education, Tehran, Iran; University of California, San Francisco, United States of America

## Abstract

**Research objective:**

This study examines the perspectives of a range of key hospital staff on the use, importance, scientific background, availability of data, feasibility of data collection, cost benefit aspects and availability of professional personnel for measurement of quality indicators among Iranian hospitals. The study aims to facilitate the use of quality indicators to improve quality of care in hospitals.

**Study design:**

A cross-sectional study was conducted over the period 2009 to 2010. Staff at Iranian hospitals completed a self-administered questionnaire eliciting their views on organizational, clinical process, and outcome (clinical effectiveness, patient safety and patient centeredness) indicators.

**Population studied:**

93 hospital frontline staff including hospital/nursing managers, medical doctors, nurses, and quality improvement/medical records officers in 48 general and specialized hospitals in Iran.

**Principal findings:**

On average, only 69% of respondents reported using quality indicators in practice at their affiliated hospitals. Respondents varied significantly in their reported use of organizational, clinical process and outcome quality indicators. Overall, clinical process and effectiveness indicators were reported to be least used. The reported use of indicators corresponded with their perceived level of importance. Quality indicators were reported to be used among clinical staff significantly more than among managerial staff. In total, 74% of the respondents reported to use obligatory indicators, while this was 68% for voluntary indicators (p<0.05).

**Conclusions:**

There is a general awareness of the importance and usability of quality indicators among hospital staff in Iran, but their use is currently mostly directed towards external accountability purposes. To increase the formative use of quality indicators, creation of a common culture and feeling of shared ownership, alongside an increased uptake of clinical process and effectiveness indicators is needed to support internal quality improvement processes at hospital level.

## Introduction

Measuring quality of care is a powerful mechanism to drive health system performance improvement. The availability of reliable quality information empowers patients to make informed decisions about where to seek health care, and supports health care providers to provide better health care. Recently, measuring quality of care in hospitals has become a main policy priority in many countries. Numerous health systems are in the process of developing and instituting requirements for routine measurement and reporting of quality data at both national and hospital levels for external and internal purposes [1]–[4]. Quality indicators can be used for summative (external) purposes to increase the accountability of hospitals towards different stakeholders (such as; government, patients or health insurers). They can also be used for formative (internal) purposes by health care organizations and providers to measure, monitor and improve the provided levels of quality of care [5], [6].

Although quality indicators represent a promising strategy for improving quality of care and accountability, the implementation process in hospitals is often faced with problems and challenges at the ground level [7]–[13]. Some of the challenges in the implementation process may originate from the absence of a role for hospital staff in the development, selection and execution of quality indicators. Successful development and implementation of quality indicators in hospitals strongly depends on frontline staff's involvement and awareness [14], [15]. In addition, the consultation with key hospital staff and their involvement in quality improvement (QI) activities may increase the effectiveness and likelihood of success of such efforts. It can provide an avenue for better communication on relevant insights into the workflow and potential challenges. It can also provide an increased sense of accountability of staff, which is positively correlated with the success of such plans in practice [10], [16]–[20].

In response to the increasing worldwide interest in quality measures, the Iranian Ministry of Health and Medical Education (MOHME) developed a set of obligatory quality indicators. These indicators have been statutorily included in the Iranian National Hospital Evaluation Program in 2002. The plan aimed to realize improvement of the quality of hospital care, support informed decision making by providing health-related information, and increasing accountability and regulation in hospitals [21]. The quality indicators in Iran were primarily designed for summative purposes to increase the accountability of hospitals for the quality of care they provide. All hospitals were obliged to implement the quality indicators applying a top-down approach steered by the MOHME. As a result, the development of the quality indicators was conducted with little consultation of hospital staff [22].

Despite nationwide efforts to develop and implement the quality indicators, at present there is no study available about the extent of application and perspective of hospital staff on such measures. This study therefore aimed to assess the perspectives of hospital frontline staff on the seven themes in regards to the selected 27 obligatory and voluntary quality indicators. The indicators are categorized in organizational, clinical process and outcome groups. The outcome indicators included clinical effectiveness, patient safety and patient centeredness issues. The themes that were assessed in this study, included: use, importance, scientific soundness, availability of data, feasibility of data collection, cost benefit aspects and availability of professional personnel for measurement of quality indicators.

## Methods

This is a cross-sectional study conducted over the period 2009 to 2010. Data was collected using a self-administered questionnaire. The survey was focused on twenty-seven hospital quality indicators, in each case exploring the perspectives of hospital frontline staff on seven themes. The indicators and themes included in the questionnaire were derived from a two-stage expert panel process. The panel of ten health experts consisted of two health services academics, two health services researchers, two hospital managers, a physician, two nurses, and an epidemiologist. To obtain the views of hospital staff on quality indicators for hospital care, it was important not to limit the selection of indicators to the set of obligatory indicators used by the MOHME. The expert panel was therefore presented with an initial list of obligatory and voluntary indicators and seven themes. The indicators and themes were selected from the current Iranian hospitals' annual evaluation program [23] and the World Health Organization Performance Assessment Tool for quality improvement in Hospitals (PATH) project [24]. In addition, the results of a literature search [25] and expert opinions on quality indicators were used. The panel was tasked with evaluating the indicators and themes, and given the opportunity to change, add or remove indicators and themes from the list.

As a result, twenty-seven quality indicators were selected by the panel. The indicators consisted of a combination of obligatory (developed by the MOHME) and voluntary indicators. Implementation of seven indicators was obligatory under the Iranian hospitals' annual evaluation program and the 20 remaining indicators recommended by the expert panel were voluntary indicators (see Table 1). The indicators were grouped based on a triad method in organizational, clinical process, and outcome indicators related to the clinical effectiveness, patient safety, and patient centeredness as shown in Table 1. The resulting questionnaire consisted of 9 organizational, 6 clinical process, and 12 outcome indicators relating to the clinical effectiveness (6), patient safety (4) and patient centeredness (2) of hospital care. The themes that were selected by the panel, included; i) the (areas of) use, ii) perceived importance of the indicator, iii) assessment of scientific background, iv) availability of data, v) feasibility of data collection, vi) cost benefit aspects, and vii) availability of professional personnel for measurement.

**Table 1:** Classification of indicators by Organizational, Clinical process, and Outcome (Obligatory and Voluntary).

Indicator type		Indicator name (O = Obligatory or V = Voluntary)	
**Organizational indicators**		Bed occupancy rate (V)	Operating room efficiency (surgical theatre use) (V)
		Length of stay (V)	Emergency room waiting time (O)
		Bed turnover (V)	Duration of quality improvement training for personnel (V)
		Wait time to admission (V)	Existence of clinical guidelines (O)
		Wait time to discharge (V)	
**Clinical process indicators**		Caesarean section rate (O)	Pre-operative antibiotic prophylaxis rate (O)
		CPR team response time (V)	Cross match rate for transfusions (V)
		Use of autologous blood rate (V)	Repeat x-ray rate in radiology (V)
**Outcome indicators**	**Clinical effectiveness**	Hospital mortality rate (V)	ICU readmission (V)
		Readmission rate (excl. day surgery) (V)	Breast feeding rate at discharge (V)
		Readmission rate after day surgery (V)	Post-operative complication rate (V)
	**Patient safety**	Needle stick injury rate (O)	Bedsore rate (V)
		Hospital-acquired infection rate (O)	Post-discharge wound infection rate (V)
	**Patient centeredness**	Patient satisfaction rate (O)	Complaint rate (V)

The questionnaire was in Farsi. It was piloted among five health services experts and ten frontline staff members (including two hospital managers, a medical doctor, two nurses, two nursing managers, two QI officers, and one head of medical records department). A purposive sample of five hospitals (three public governmental, one Social Security Organization (SSO), and one private hospital) was selected for piloting, which were not included in the sample of final survey. The questionnaire was assessed for face validity and clarity based on the responses received.

The questionnaire was self-administered with the exception of a few respondents in Tehran, capital city of Iran, whose questionnaires were administered face-to-face. The questionnaires were sent out and returned by mail with support provided for the respondents by email and telephone. Participation in the survey was voluntary and periodic reminders were sent out. Questionnaires received were assessed for completeness and consistency. Respondents were contacted to address any observed issues. Questionnaires were excluded if these issues could not be resolved.

In the final questionnaire, the perspectives of hospital frontline staff were asked on the organizational, clinical process and outcome quality indicators in relation to the seven themes. We analyzed the heterogeneity in the responses of staff to the questions by conducting Pearson's chi-squared test using SPSS. We explored whether hospital staff (both total study population, and different types of professionals) had statistically significant different perspectives on the seven themes related to the implementation of specific quality indicators. The average rates of positive answers to questions on the use, level of importance, scientific background, data and personnel availability, feasibility of data collection and cost benefit aspects of quality indicators were used in the analysis (i.e. whether there is agreement in the responses). Our criterion for the significance level was p<0.05.

### Study population

The study population consisted of 160 respondents in three groups; managerial staff (active hospital managers, quality improvement officers, the heads of medical records department), clinical staff (medical doctors, nurses and nursing managers), and other health professionals (e.g. health economists and health services researchers). They were working in 75 hospitals across nine regions in Iran during the study period. The 75 hospitals were selected using a purposive sampling method, representing (around) 10% of hospitals in Iran. The studied hospitals were largely representative in terms of type (public, private, SSO, and other – military and charity – hospitals) and geographical distribution.

The response rate of hospital staff was 71.3% (114 respondents out of 160 sampled in the study) and covered 64% (48) of the (75) sampled hospitals in the study. Twenty-one questionnaires were excluded for reasons of incompleteness or inconsistency, resulting in a final population of 93 respondents. Table 2 shows the characteristics of the study population, which is representative both across hospitals and type of professionals.

**Table 2:** Characteristics of the study population.

Hospitals						
Ownership		Sampled n (%)	Respondents n (%)	Non-respondents n (%)	Included in the study analysis n (%)	Excluded from the study analysis n (%)
Public (governmental)		48 (64)	37 (71)	11 (48)	37 (77)	-
Private		12 (16)	10 (19)	2 (9)	7 (15)	3 (75)
SSO		5 (7)	2 (4)	3 (13)	2 (4)	-
Other (Charity and/or military hospitals)		10 (13)	3 (6)	7 (30)	2 (4)	1 (25)
**Total**		**75 (100)**	**52 (100)**	**23 (100)**	**48 (100)**	**4 (100)**
**Staff**						
**Job description**		**Sampled n (%)**	**Respondents n (%)**	**Non-respondents n (%)**	**Included in the study analysis n (%)**	**Excluded from the study analysis n (%)**
Managerial staff	Hospital manager	30 (19)	18 (16)	12 (26)	14 (15)	4 (19)
	Quality improvement officer	27 (17)	23 (20)	4 (9)	15 (16)	8 (38)
	Medical records officer	31 (19)	25 (22)	6 (13)	20 (21)	5 (24)
	**Sub-total**	**88 (55)**	**66 (58)**	**22 (48)**	**49 (53)**	**17 (81)**
Clinical staff	Doctor or nurse	20 (13)	13 (11)	7 (15)	10 (11)	3 (14)
	Nursing manager*	37 (23)	28 (25)	9 (19)	28 (30)	-
	**Sub-total**	**57 (36)**	**41 (36)**	**16 (34)**	**38 (41)**	**3 (14)**
Other staff (Relevant professionals)	Health economist	3 (2)	1 (1)	2 (4)	1 (1)	-
	Health services managers	5 (3)	3 (2)	2 (4)	2 (2)	1 (5)
	Medical engineer	2 (1)	1 (1)	1 (3)	1 (1)	-
	Industrial manager	2 (1)	1 (1)	1 (3)	1 (1)	-
	Insurance manager	3 (2)	1 (1)	2 (4)	1 (1)	-
	Sub-total	15 (9)	7 (6)	8 (18)	6 (6)	1 (5)
**Total**		**160 (100)**	**114 (100)**	**46 (100)**	**93 (100)**	**21 (100)**

* Including matron, teaching supervisor, and clinical supervisor.

The study was approved by the Deputy of Research and Technology of the Tehran (Iran) University of Medical Sciences (Code: 958/1635996).

## Results

### Perspectives of hospital staff on quality indicators

#### Quality indicators in general

**Figure 1:**
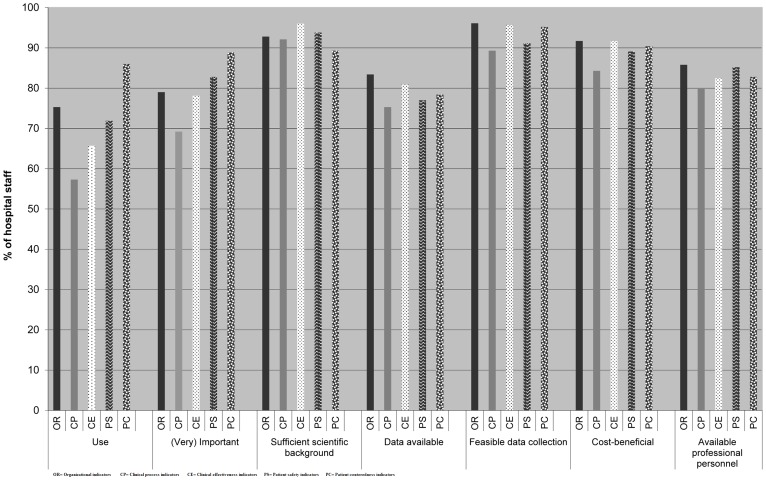
Overall perspectives of hospital staff on organizational, clinical process, and outcome indicators. Figure 1 shows the reported utilization rates for each of the indicator groups, which were: organizational indicators (75%), clinical process indicators (57%), clinical effectiveness indicators (66%), patient safety indicators (72%), and patient centeredness indicators (86%). Around 80% of respondents valued the indicators as (very) important and more than 89% of them reported sufficient scientific background for indicators. Data were reported to be available for indicators by at least 75% of respondents. Data collection was judged to be feasible by 96%, 89%, and 94% of respondents for organizational, clinical process and outcome indicators respectively. However, the availability of personnel and cost benefit aspects of indicators was judged to be sufficient by more than 80% of respondents. In figure 1: OR =  Organizational indicators CP =  Clinical process indicators CE =  Clinical effectiveness indicators PS =  Patient safety indicators PC =  Patient centeredness indicators

Hospital staff had significantly different (heterogeneous) perspectives on the use, level of importance, scientific background, feasibility of data collection, and cost benefit aspects of measurement of quality indicators. In contrast, there was more agreement (homogeneity) in the perspectives of hospital staff on the availability of data and professional personnel to measure the indicators. Figure 1 summarizes the overall perspectives of respondents. Organizational, clinical process, clinical effectiveness, patient safety, and patient centeredness indicators were reported to be used by 75%, 57%, 66%, 72%, and 86% of the respondents respectively (p<0.05). Clinical process indicators had the lowest reported use. This was also the indicator group with the lowest perceived importance by hospital staff. The importance given to indicators corresponded with their use, which was particularly high for outcome indicators compared to the other type of quality indicators (p<0.05). Despite the sufficient scientific background reported by respondents, on average, only 68% of respondents reported using quality indicators at their affiliated hospitals. Data were thought to be available by 83% of respondents for organizational indicators, and only 75% and 79% of respondents thought data was available for the clinical process and outcome indicators respectively. Data collection was judged to be feasible by 96%, 89%, and 94% of respondents for organizational, clinical process, and outcome indicators respectively. However, the availability of personnel and cost benefit aspects of organizational, clinical process, and outcome indicators was judged to be sufficient by 86%, 80%, 84%, and 92%, 84%, 91% of respondents respectively.

#### Organizational indicators

Table 3 shows the responses to the questions related to the organizational indicators. Respondents had significantly different perspectives on the level of importance, scientific background, data and professional personnel availability of the organizational indicators. However, they did not have significant different perspectives on the use, feasibility of data collection and cost benefit aspects of these indicators. The most commonly used measures were the bed occupancy rate, length of stay and bed turnover (p<0.05). The operating room (OR) efficiency indicator had a significantly lower reported use in this group, followed by a measure of the duration of quality assurance training. Medium scoring (around 73% reported use) indicators in this group were related to waiting times. Lower use of organizational indicators seemed to be mostly related to the perceived importance, availability of data, and available human resources to collect the data.

**Table 3:** The perspectives of hospital staff on organizational indicators (Obligatory and Voluntary).

Indicator	Bed occupancy rate (V) % (n)	Length of stay (V) % (n)	Bed turnover (V) % (n)	Wait time to admission (V) % (n)	Wait time to discharge (V) % (n)	OR efficiency (V) % (n)	Emergency room waiting time (O) % (n)	Duration of quality assurance training (V) % (n)	Existence of clinical guidelines (O) % (n)	P-value
Perspective										
**Do you use this indicator?**										0.091
No	7 (6)	10 (9)	13 (12)	17 (16)	19 (18)	28 (25)	19 (17)	21 (19)	10 (9)	
Yes	89 (83)	88 (82)	79 (71)	74 (69)	72 (67)	54 (49)	73 (67)	64 (58)	84 (77)	
Don't know	4 (4)	2 (2)	8 (7)	9 (8)	9 (8)	18 (16)	9 (8)	15 (14)	7 (6)	
**If yes, where**										
External assessment	61 (57)	67 (62)	47 (44)	41 (38)	38 (35)	30 (25	34 (32)	33 (31)	44 (41)	
Internal Audit	52 (48)	46 (43)	40 (37)	43 (40)	39 (36)	28 (26)	39 (36)	33 (31)	47 (44)	
Surprise inspection	23 (21)	19 (18)	19 (18)	22 (20)	14 (13)	9 (8)	17 (16)	16 (15)	32 (30)	
Planned inspection	32 (30)	24 (22)	23 (21)	18 (17)	12 (11)	13 (12)	20 (19)	18 (17)	22 (20)	
Peer Review	22 (20)	22 (20)	16 (15)	16 (15)	14 (13)	15 (14)	15 (14)	18 (17)	22 (20)	
Other	4 (4)	5 (5)	4 (4)	8 (7)	7 (6)	2 (2)	2 (2)	3 (3)	7 (6)	
**Do you think this indicator is important?**										0.000
Not Important	0 (0)	0 (0)	0 (0)	0 (0)	0 (0)	0 (0)	0 (0)	1 (1)	0 (0)	
A little Important	1 (1)	5 (4)	4 (3)	4 (3)	4 (3)	8 (5)	0 (0)	3 (2)	4 (3)	
Moderately important	16 (14)	13 (11)	17 (14)	19 (15)	22 (16)	28 (18)	20 (15)	11 (8)	15 (12)	
Important	38 (34)	42 (37)	31 (25)	35 (27)	32 (24)	42 (27)	31 (23)	37 (27)	33 (27)	
Very Important	45 (40)	41 (36)	48 (39)	42 (33)	42 (31)	23 (15)	49 (36)	48 (35)	49 (41)	
**Do you think this indicator has a sufficient scientific background?**										0.047
No	5 (4)	6 (5)	5 (4)	11 (9)	9 (7)	10 (7)	10 (7)	6 (4)	5 (4)	
Yes	96 (85)	94 (83)	95 (79)	89 (71)	91 (71)	90 (60)	90 (66)	94 (67)	95 (77)	
**Are the data available for this indicator?**										0.045
No	1 (1)	2 (2)	2 (2)	14 (11)	12 (9)	3 (2)	14 (10)	16 (11)	12 (9)	
Yes	98 (86)	93 (80)	92 (76)	77 (61)	81 (61)	82 (51)	79 (55)	68 (46)	75 (58)	
Don't know	1 (1)	5 (4)	6 (5)	9 (7)	7 (5)	15 (9)	7 (5)	16 (11)	13 (10)	
**Is it feasible to collect the data for this indicator?**										0.216
No	0 (0)	0 (0)	4 (3)	4 (3)	3 (2)	5 (3)	4 (3)	7 (5)	10 (8)	
Yes	100 (88)	100 (87)	96 (79)	96 (74)	97 (73)	96 (64)	96 (67)	93 (66)	90 (72)	
**Is measuring this indicator beneficial given the costs?**										0.585
No	5 (4)	4 (3)	9 (7)	10 (8)	4 (3)	12 (8)	10 (7)	12 (8)	13 (10)	
Yes	95 (82)	97 (83)	92 (75)	90 (71)	96 (74)	88 (58)	90 (65)	88 (61)	88 (70)	
**Are professional personnel available to measure this indicator?**										0.005
No	5 (4)	7 (6)	7 (6)	17 (13)	16 (12)	16 (11)	20 (14)	17 (12)	27 (22)	
Yes	95 (83)	93 (80)	93 (77)	83 (65)	84 (64)	84 (56)	80 (57)	83 (60)	73 (60)	

#### Clinical process indicators

Respondents assessed the use, level of importance, scientific background, data and professional personnel availability of the clinical process indicators significantly different. More agreement existed on the feasibility of data collection and cost benefit aspects of these indicators. Table 4 shows that clinical process indicators were reported to be significantly less used compared to the organizational and outcome indicators. In addition, there was also a relatively high level of unawareness among staff (19%) whether or not the clinical process indicators were applied in their affiliated hospitals, compared to the organizational (9%) and outcome (11%) indicators. On the extreme, only 38% of respondents reported to apply cross match rates for transfusions (and 26% of respondents were unaware of the use of this indicator). The indicators were mostly reported to be used for external assessments and internal audits purposes.

**Table 4:** The perspectives of hospital staff on clinical process indicators (Obligatory and Voluntary).

Indicator	Caesarean section rate (O) % (n)	CPR team response time (V) % (n)	Use of autologous blood rate (V) % (n)	Pre-operative antibiotic prophylaxis rate (O) % (n)	Cross match rate for transfusions (V) %(n)	Repeat x-ray rate in radiology (V) % (n)	P-value
Perspective							
**Do you use this indicator?**							0.010
No	20 (17)	24 (22)	16 (15)	25 (22)	37 (33)	24 (22)	
Yes	67 (83)	55 (82)	73 (71)	53 (69)	38 (67)	58 (53)	
Don't know	13 (11)	21 (19)	11 (10)	23 (20)	26 (23)	19 (17)	
**If yes, where**							
External assessment	41 (38)	32 (30)	30 (28)	26 (24)	15 (14)	22 (20)	
Internal Audit	33 (31)	26 (24)	39 (36)	24 (22)	23 (21)	32 (30)	
Surprise inspection	15 (14)	16 (15)	25 (23)	9 (8)	8 (7)	12 (11)	
Planned inspection	19 (18)	11 (10)	16 (15)	10 (9)	7 (6)	15 (14)	
Peer Review	13 (12)	11 (10)	11 (10)	14 (13)	8 (7)	20 (19)	
Other	4 (4)	4 (4)	4 (4)	1 (1)	3 (3)	3 (3)	
**Do you think this indicator is important?**							0.000
Not Important	6 (4)	0 (0)	0 (0)	2 (1)	4 (2)	3 (3)	
A little Important	3 (2)	3 (2)	1 (1)	11 (7)	12 (6)	10 (7)	
Moderately important	15 (10)	26 (17)	19 (14)	24 (15)	27 (14)	22 (15)	
Important	38 (26)	29 (19)	28 (20)	23 (14)	19 (10)	31 (21)	
Very Important	39 (27)	42 (27)	51 (37)	40 (25)	39 (20)	33 (22)	
**Do you think this indicator has a sufficient scientific background?**							0.035
No	7 (5)	8 (5)	7 (5)	11 (7)	9 (5)	6 (4)	
Yes	93 (64)	92 (61)	93 (68)	89 (55)	91 (52)	94 (62)	
**Are the data available for this indicator?**							0.004
No	2 (1)	7 (5)	13 (9)	17 (10)	17 (9)	24 (15)	
Yes	99 (66)	71 (49)	78 (54)	72 (42)	69 (37)	60 (38)	
Don't know	0 (0)	22 (15)	9 (6)	10 (6)	15 (8)	16 (10)	
**Is it feasible to collect the data for this indicator?**							0.148
No	3 (2)	9 (6)	6 (4)	11 (7)	16 (9)	21 (13)	
Yes	97 (65)	91 (60)	94 (65)	89 (55)	84 (47)	79 (50)	
**Is measuring this indicator beneficial given the costs?**							0.584
No	8 (5)	13 (9)	9 (6)	23 (14)	24 (13)	21 (13)	
Yes	92 (61)	87 (59)	92 (65)	77 (48)	76 (42)	79 (48)	
**Are professional personnel available to measure this indicator?**							0.008
No	9 (6)	20 (13)	17 (12)	22 (13)	30 (17)	27 (16)	
Yes	91 (63)	80 (53)	83 (60)	78 (47)	70 (39)	73 (43)	

#### Outcome indicators

Table 5 shows the results for the questions on outcome indicators related to clinical effectiveness, patient safety and patient centeredness. Hospital staff had significantly different perspectives on the use, level of importance, data and professional personnel availability of the clinical effectiveness outcome indicators. However, they had relatively similar opinions on the scientific background, feasibility of data collection and cost benefit aspect of these indicators. Significant heterogeneity existed in the responses of hospital staff on the use, level of importance, data and professional personnel availability, and feasibility of data collection of the patient safety outcome indicators. More homogenous responses were reported regarding the scientific background and cost benefit aspect of these indicators. Hospital staff also significantly differed in their perspectives on the availability of data and professional personnel with regard to the patient centeredness outcome indicators. In contrast, such heterogeneity was not seen regarding the use, level of importance, scientific background, feasibility of data collection, and cost benefit aspect of these indicators.

**Table 5:** The perspectives of hospital staff on outcome indicators (related to clinical effectiveness, patient safety and patient centeredness; Obligatory and Voluntary).

Indicators	Clinical effectiveness outcome indicators						P-value	Patient safety outcome indicators				P- value	Patient centeredness outcome indicators		P-value
Perspective	Hospital mortality rate (V) % (n)	Re-admission rate (excl. day surgery) (V) % (n)	Re-admission rate after day surgery (V) % (n)	ICU re-admission rate (V) % (n)	Breast feeding rate at discharge (V) % (n)	Post-operative complication rate (V) % (n)		Needle stick injury rate (O) % (n)	Post-discharge wound infection rate (V) % (n)	Hospital acquired infection rate (O) % (n)	Bed-sore rate (V) % (n)		Patient satisfaction rate (O) % (n)	Complaint rate (V) % (n)	
**Do you use this indicator?**							0.001					0.012			0.639
No	9 (8)	21 (19)	26 (24)	25 (23)	33 (28)	21 (19)		21 (19)	24 (22)	4 (4)	17 (15)		9 (8)	10 (9)	
Yes	91 (85)	63 (58)	56 (51)	55 (50)	57 (48)	71 (64)		62 (57)	60 (55)	90 (84)	75 (68)		88 (82)	84 (78)	
Don't know	0 (0)	16 (15)	18 (16)	20 (18)	11 (9)	8 (7)		17 (16)	15 (14)	5 (5)	9 (8)		3 (3)	7 (6)	
**If yes, where**															
External assessment	69 (64)	33 (31)	30 (28)	25 (23)	24 (22)	34 (32)		30 (28)	29 (27)	66 (61)	31 (29)		59 (55)	46 (43)	
Internal Audit	50 (46)	32 (30)	24 (22)	26 (24)	31 (29)	38 (35)		32 (30)	39 (36)	61 (57)	42 (39)		52 (48)	53 (49)	
Surprise inspection	27 (25)	14 (13)	12 (11)	13 (12)	16 (15)	16 (15)		17 (16)	22 (20)	36 (33)	23 (21)		29 (27)	28 (26)	
Planned inspection	30 (28)	13 (12)	10 (9)	11 (10)	14 (13)	20 (19)		13 (12)	19 (18)	36 (33)	19 (18)		27 (25)	29 (27)	
Peer Review	22 (20)	22 (20)	15 (14)	12 (11)	16 (15)	20 (19)		11 (10)	18 (17)	27 (25)	20 (19)		19 (18)	22 (20)	
Other	8 (7)	5 (5)	4 (4)	7 (6)	3 (3)	3 (3)		4 (4)	7 (6)	4 (4)	3 (3)		3 (3)	4 (4)	
**Do you think this indicator is important?**							0.000					0.000			0.229
Not Important	0 (0)	1 (1)	2 (1)	0 (0)	3 (2)	0 (0)		0 (0)	0 (0)	0 (0)	0 (0)		0 (0)	0 (0)	
Less Important	1 (1)	4 (3)	6 (4)	5 (3)	6 (4)	4 (3)		7 (5)	8 (5)	2 (2)	3 (2)		2 (2)	4 (3)	
Moderately	11 (10)	11 (8)	22 (14)	28 (17)	20 (13)	12 (9)		13 (9)	15 (10)	9 (8)	15 (12)		5 (4)	12 (10)	
Important	25 (22)	41 (30)	34 (22)	31 (19)	30 (19)	29 (21)		43 (31)	28 (19)	20 (18)	30 (24)		31 (26)	33 (28)	
Very Important	63 (56)	43 (32)	36 (23)	36 (22)	41 (26)	55 (40)		38 (27)	49 (33)	69 (62)	52 (41)		62 (53)	52 (44)	
**Do you think this indicator has a sufficient scientific background?**							0.336					0.057			0.299
No	1 (1)	6 (4)	5 (3)	3 (2)	5 (3)	6 (4)		9 (6)	9 (6)	0 (0)	9 (7)		7 (6)	14 (12)	
Yes	99 (89)	95 (69)	95 (61)	97 (61)	96 (63)	94 (67)		92 (65)	91 (61)	100 (91)	91 (72)		93 (78)	86 (73)	
**Are the data available for this indicator?**							0.029					0.001			0.032
No	3 (3)	10 (7)	8 (5)	10 (6)	9 (6)	10 (7)		13 (9)	15 (10)	8 (7)	9 (7)		12 (10)	7 (6)	
Yes	97 (85)	78 (57)	79 (50)	78 (49)	74 (48)	75 (53)		71 (51)	70 (46)	82 (73)	83 (64)		76 (64)	81 (67)	
Don't know	0 (0)	12 (9)	13 (8)	13 (8)	17 (11)	16 (11)		17 (12)	15 (10)	10 (9)	8 (6)		12 (10)	12 (10)	
**Is it feasible to collect the data for this indicator?**							0.801					0.040			0.106
No	0 (0)	8 (6)	3 (2)	3 (2)	5 (3)	7 (5)		9 (6)	15 (10)	9 (8)	4 (3)		4 (3)	6 (5)	
Yes	100 (89)	92 (67)	97 (61)	97 (61)	95 (62)	93 (65)		92 (65)	85 (57)	91 (81)	96 (72)		96 (80)	94 (79)	
**Is measuring this indicator beneficial given the costs?**							0.260					0.057			
No	1 (1)	8 (6)	7 (4)	13 (8)	11 (7)	13 (9)		8 (6)	17 (11)	9 (8)	11 (8)		7 (6)	12 (10)	
Yes	99 (88)	92 (66)	94 (58)	87 (54)	89 (57)	87 (62)		92 (66)	83 (55)	91 (82)	90 (68)		93 (77)	88 (74)	
**Are professional personnel available to measure this indicator?**							0.001					0.006			0.028
No	1 (1)	29 (21)	25 (16)	21 (13)	12 (8)	22 (15)		13 (9)	24 (16)	12 (11)	12 (9)		16 (13)	19 (16)	
Yes	99 (87)	71 (51)	75 (47)	79 (50)	88 (59)	78 (54)		87 (62)	76 (50)	88 (80)	88 (67)		85 (71)	81 (69)	

### Perspectives of managerial, clinical and other hospital staff on different types of quality indicators

**Figure 2:**
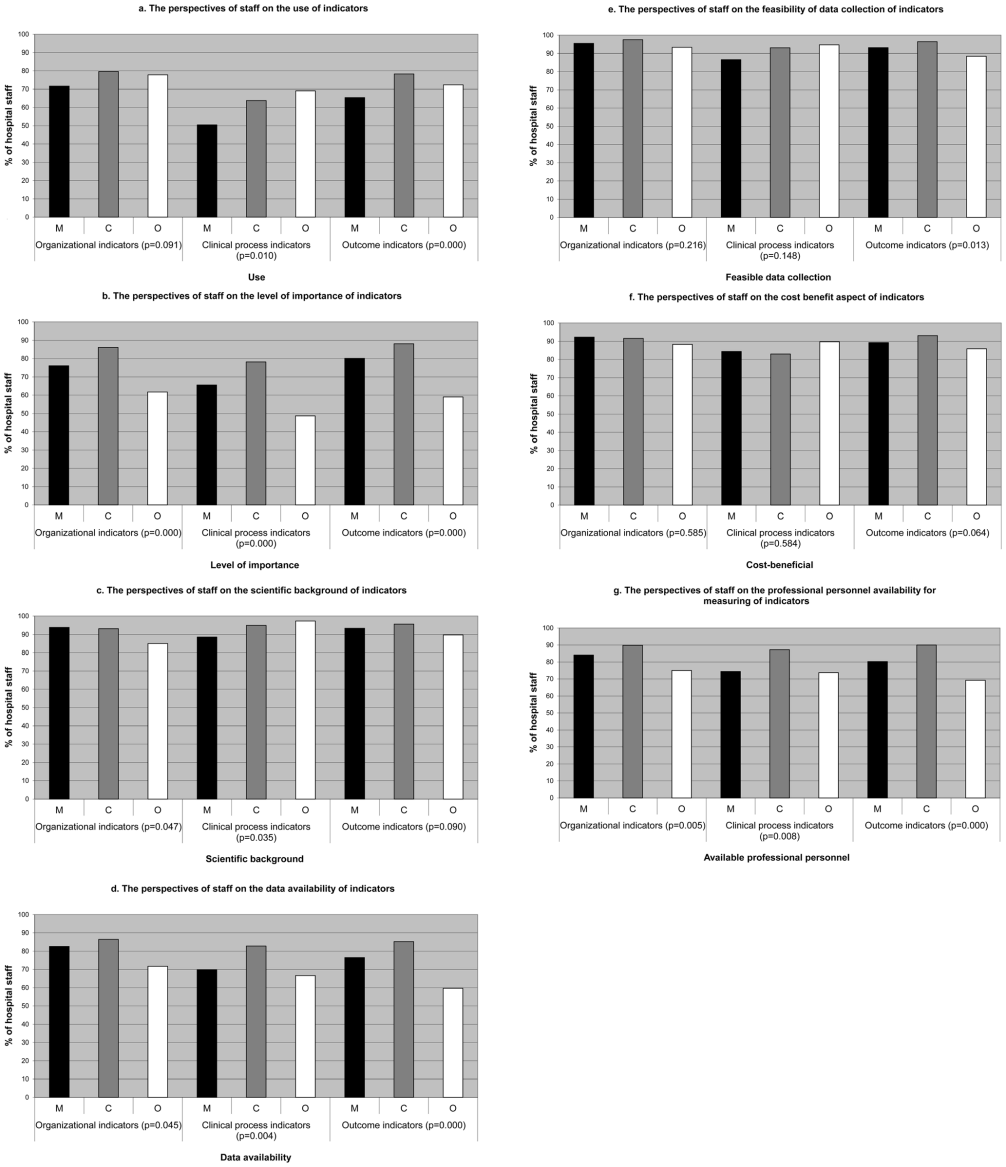
The perspectives of managerial, clinical and other staff on organizational, clinical process, and outcome quality indicators. Figure 2 shows that clinical staff reported significantly higher utilization rates than managerial staff, i.e.: organizational indicators (80% versus 71%), clinical process indicators (64% versus 51%), clinical effectiveness indicators (75% versus 58%) and patient safety indicators (81% versus 66%). Both groups reported more or less equal use of patient centeredness indicators. There were about equally large (significant) differences among managerial and clinical staff in the level of perceived importance of indicators. However, both groups rated organizational and clinical outcome indicators as most important. Both clinical and managerial staff rated the scientific background of all indicators relatively high. Clinical staff rated data availability for the organizational, clinical process, and outcome indicators significantly higher than managerial staff..Professional personnel for measuring organizational, clinical process and outcome indicators were thought to be available by 90%, 87% and 90% of clinical staff versus 84%, 74% and 80% by managerial staff. In figure(s) 2: M =  Managerial staff C =  Clinical staff O =  Other staff

Figure 2 shows the perspectives of managerial, clinical and other staff on organizational, clinical process and outcome indicators. The corresponding p-values indicate the statistical differences in perspectives of staff on each indicator. Clinical staff reported significantly higher utilization rates than managerial staff, i.e.: organizational indicators (80% versus 71%), clinical process indicators (64% versus 51%), clinical effectiveness indicators (75% versus 58%) and patient safety indicators (81% versus 66%). Both groups reported more or less equal use of patient centeredness indicators. The differences in reported use were (significantly) higher in case of clinical process, clinical effectiveness and patient safety indicators, compared to organizational indicators. There were about equally large (significant) differences among managerial and clinical staff in the level of perceived importance of indicators. However, both groups rated organizational and clinical outcome indicators as most important. Both clinical and managerial staff rated the scientific background of all indicators relatively high.

Clinical staff rated data availability significantly higher than managerial staff, i.e.: organizational indicators (87% versus 83%), clinical process indicators (83% versus 70%), and outcome indicators (85% versus 77%). The same was true for the perspectives on the feasibility of data collection and availability of professional personnel.

### Perspectives on obligatory and voluntary indicators

Hospital staff had significantly different perspectives on the use, level of importance, scientific background, data availability, feasibility of data collection and professional personnel availability on the obligatory and voluntary indicators. However, there was no significant difference in the perspectives of staff on the cost benefit aspect of the obligatory and voluntary indicators. In total, 74% of respondents reported using obligatory indicators for different processes in their hospital. Sixty-eight percent of the respondents reported use of the remaining twenty voluntary indicators. Although the implementation of seven indicators in our study was obligatory as part of external evaluation program, only 44% of respondents reported using these indicators in the hospitals.

## Discussion

We studied the perspective of frontline staff on the seven themes related to the implementation of 27 quality indicators in Iranian hospitals. This is to our knowledge the first study providing insight into the perspectives of frontline hospital staff on important aspects related to the use and implementation of quality indicators in Iranian hospitals. The comprehensive scope of the included (consensus-based) quality indicators, representative sample of all types of hospitals, and breadth of hospital professionals are strong features of the study design. However, the study has some limitations. First, the non-response of 28.7% of hospital staff and exclusion of around 20% of received questionnaires from our study population due to incompleteness of surveys resulted in a lower response rate than planned. It is possible that the non-represented hospitals had a lower awareness of the importance and usability of quality indicators, leading to a potential (over-)reporting bias. Furthermore, there was no clear standardized classification of quality indicators available for our study. This may be explained by the various definitions of quality indicators used by different users. However, the classification of indicators studied in this survey was based on relevant literature, knowledge and the experience of the authors.

Our survey shows that currently, hospital staff are primarily applying organizational (75%) and clinical outcome (71%) indicators. Only 57% of staff reported using the clinical process indicators which is significantly lower than their reported use of organizational and outcome indicators. The reported use corresponded with the perceived level of importance of indicators. There is a significant difference in the responses of hospital staff on the use, level of importance, feasibility of data collection, scientific background, and cost benefit aspects of measurement of the indicators. More agreement existed among hospital staff on the availability of data and professional personnel to measure the indicators. Clinical staff reported a significantly higher use of all types of indicators (except patient centeredness) compared to managerial staff. There were statistically significant differences in the reported use of obligatory and voluntary indicators. Not all obligatory quality indicators were applied by respondents. Only 74% of respondents reported to apply obligatory indicators, while 68% reported to use voluntary indicators.

### Ensuring availability of dedicated staff for measuring quality indicators

Despite the acknowledgement of the importance and scientific background of quality indicators, just around two-third of hospital staff in total reported using quality indicators at practice level in their hospitals. The overall application rate shows an interest in the use of quality indicators in Iranian hospitals. However, we found significant differences between the rate of use in daily practice and the perceived importance and scientific background of different quality indicators by hospital staff. The results indicate a gap between theory and practice in the utilization of quality indicators by hospital frontline staff. Hospital staff believe that quality indicators are sufficiently grounded in science and should play an important role for monitoring quality and quality improvement (also reported by other studies [26], [27]) in hospitals. However, they experienced a number of problems in the implementation of quality indicators in practice. The results of our study show that the availability of dedicated staff for measuring indicators is one of the major concerns for using quality indicators from the perspective of hospital frontline staff. This was also confirmed by related studies in this field [10], [16]. For health care professionals to actually use quality indicators it is essential to involve hospital staff in the development and implementation of indicators. Other studies also identified that the involvement and participation of staff is associated with the success of quality improvement initiatives and better values of quality indicators in hospitals [10], [28], [29].

### More attention on clinical process and effectiveness indicators

Our comparison of the overall utilization of different quality indicators revealed that there is significant difference between organizational, outcome and clinical process indicators. Although more than two-thirds of respondents acknowledged using the organizational and outcome indicators, only around half (57%) of them reported using the clinical process indicators at their hospitals. In addition, when looking at the type of outcome indicators, the results showed that clinical effectiveness indicators were used significantly less than the patient safety and patient centeredness indicators. Clinical process and effectiveness indicators seem to provide a current challenge in Iranian hospitals. The lower application rate of clinical process and effectiveness indicators in Iranian hospitals can be explained by the insufficient transparency to staff (including clinicians). Moreover, lower involvement of staff in the development process and a lack of awareness of the relevance of clinical process indicators are other possible factors resulting to a lower implementation rate. Related studies also identified less utilization of clinical indicators compared to organizational and outcome indicators in hospitals. They identified difficulties in developing evidence-based clinical process and effectiveness indicators and being less transparent to patients and staff as main barriers in the use of such measures in hospitals. In addition, they found the fear of punitive measures for variation in clinical processes and a lack of awareness or familiarity of staff with quality indicators as other possible problems in the use of these indicators [13], [29], [30].

Our study results indicate a limited use of effectiveness indicators, but also clinical process indicators whose actionability is important for QI purposes. Where organizational indicators are helpful to set the right conditions for providing high quality care, outcome indicators are very attractive for both accountability and informative purposes. In addition, they can be use to monitor the achieved levels of quality of care in hospitals. However, when outcome indicators show negative results, one needs clinical process indicators to locate the causes of reduced quality levels to improve the work processes. At the moment the interest in Iran both among clinicians and hospital managers appears to be overly focused on organizational and outcome indicators. To stimulate QI activities at hospital level, it is necessary that both clinicians and hospital managers work with clinical process and effectiveness indicators alongside organizational, patient safety and patient centeredness outcome indicators.

### Combining summative with formative purposes of quality indicators

Our study shows that the current quality indicators system in Iran (which has been primarily set up for summative purposes to increase the accountability), may not be used for formative purposes in practice. Our results show that despite the government's obligation to apply quality indicators, at best, only two-thirds of respondents reported to use such measures in practice. This rate points to barriers in the implementation of quality measures by hospitals. It is perhaps the result of a low awareness among frontline staff and lack of involvement in the development of quality indicators. Staff's low engagement, lack of time, limited awareness and knowledge concerning quality indicators can lead to a low motivation and commitment in applying such measures in practice [14], [28], [29]. The results suggest that having a top-down implementation method may not be sufficient to achieve a maximum expected implementation and effective application of quality indicators. This needs to be complemented with a bottom up approach, involvement and widespread participation of hospital staff and encouragement to participate in QI activities to create a facilitating quality improvement culture [10], [22], [28], [29].

### Creating a common quality improvement culture

We found that clinical and managerial staff had different views on the use, level of importance, scientific background, feasibility of data collection and cost benefit aspects of organizational, clinical process and outcome indicators. Clinical staff on average reported using quality indicators more than managerial staff (with the exception of patient centeredness indicators). The possible reasons for such differences may be different perspectives of staff on the level of importance of indicators, data and professional personnel availability for the specific indicators. Clinical staff believe that quality indicators are very important for QI in hospital, while managerial staff did not share this opinion and even around 4% of managerial staff mentioned that clinical process indicators are not important at all in the QI process in hospitals. The opinion of hospital staff can be changed if they feel more capable in monitoring and improving their own quality of services. This starts by involving them in the development phase of quality indicators, and selection of implementation strategy at hospital level. A feeling of increased autonomy, shared ownership and awareness will likely be beneficial for the actual (and appropriate) use of quality indicators [29]. However, this also requires a hospital culture characterized by learning, constructive feedback and discussion among professionals about health care performance without creating a punitive environment. Though essential, this is challenging to create.

## Conclusions

Although there is a general awareness of the importance and usability of quality indicators among hospital staff in Iran, currently, their use is mostly directed towards external accountability purposes. The impact of quality indicators may be increased by extending the use of clinical process and effectiveness indicators in addition to organizational, patient safety and patient centeredness outcome indicators. It is important to explore options for increasing the formative use of quality indicators to support internal quality improvement processes at hospital level. For this, the indicators will need to become part of the working methods of hospital staff to create a culture and feeling of shared ownership for the quality indicator system.
